# Challenges in measuring interprofessional–interorganisational collaboration with a questionnaire

**DOI:** 10.3399/bjgpopen18X101385

**Published:** 2018-04-21

**Authors:** Loes J Meijer, Esther de Groot, Maarten van Smeden, François G Schellevis, Roger AMJ Damoiseaux

**Affiliations:** 1 GP and PhD Student, Department of GP Training, Julius Center for Health Sciences and Primary Care, UMC Utrecht, Utrecht, The Netherlands; 2 Assistant Professor, Department of GP Training, Julius Center for Health Sciences and Primary Care, UMC Utrecht, Utrecht, The Netherlands; 3 Statistician and Senior Researcher in Epidemiology and Medical Statistics, Department of Biostatistics and Research Support, Julius Center for Health Sciences and Primary Care, UMC Utrecht, Utrecht, The Netherlands; 4 Professor of Primary Care, Department of Primary Care, NIVEL (Netherlands Institute for Health Services Research), Utrecht, The Netherlands; 5 Professor, Department of General Practice & Elderly Care Medicine, Amsterdam Public Health Research Institute, VU University Medical Centre, Amsterdam, The Netherlands; 6 Professor of Primary Care, Department of GP Training, Julius Center for Health Sciences and Primary Care, UMC Utrecht, Utrecht, The Netherlands

**Keywords:** primary care, secondary care, interprofessional, interdisciplinary collaboration, questionnaire

## Abstract

**Background:**

Collaboration between medical professionals from separate organisations is necessary to deliver good patient care. This care is influenced by professionals’ perceptions about their collaboration. Until now, no instrument to measure such perceptions was available in the Netherlands. A questionnaire developed and validated in Spain was translated to assess perceptions about clinicians’ collaboration in primary and secondary care in the Dutch setting.

**Aim:**

Validation in the Dutch setting of a Spanish questionnaire that aimed to assess perceptions of clinicians about interorganisational collaboration.

**Design & setting:**

After translation, cultural adaptation, and pre-testing, the questionnaire was sent to GPs and secondary care clinicians (SCCs) in three regions in the Netherlands. The responses of 445 responders were used to assess the validity and reliability of the questionnaire.

**Method:**

A confirmatory factor analysis (CFA) and an exploratory factor analysis (EFA) were performed to study the construct validity of the hypothesised factor model underlying the questionnaire. Test-retest reliability was evaluated using weighted Kappa statistics.

**Results:**

Results of the CFA indicated poor fit of the hypothesised factor structure. EFA, executed separately for each region, showed a highly unstable factor structure. The test-retest reliability analysis demonstrated low re-test reliability.

**Conclusion:**

The underlying factor structure of a Spanish questionnaire could not be reproduced. The construct validity and reliability of this questionnaire were insufficient to warrant use in the Dutch setting. This study demonstrates the need for evaluating validity and reliability of questionnaires in local settings.

## How this fits in

A validated Spanish questionnaire was found to be not valid in the Netherlands. When measuring professionals’ perceptions about collaboration, one needs to be aware that a questionnaire developed and validated in other settings is not to be used lightly in a different context. Evaluating validity and reliability of such questionnaires in local settings is essential.

## Introduction

Interprofessional collaboration is necessary for patient-centred care, especially in the complex context of an ageing population in which multimorbidity is common.^1^ Patients who suffer from multimorbidity often receive care from multiple healthcare professionals, and frequently undergo transitions between healthcare organisations and settings. When professionals from different organisations contribute to patient care, ensuring continuity of care is important.^2–6^ The transitions of patients, in particular when moving from the primary care setting to secondary care, benefit significantly from good collaboration between GPs and SCCs, and between the organisations in which they work.^6,7^ Even when a healthcare professional functions well on their own, patient care can be suboptimal when professionals work in a fragmented healthcare system with many boundaries between primary care, outpatient settings, and hospitals.^7–14^

In the Netherlands, as in many other countries, various initiatives are undertaken to improve collaborative patient care across organisations.^10,15^ Whether these efforts to improve the conditions for collaboration on an individual and an organisational level are successful often remains unclear because adequate instruments to measure collaboration are lacking. Only a few questionnaires have been developed which aim to measure the level of collaboration of professionals in a single organisation or a single team.^16–20^ Questionnaires that measure conditions for collaboration across the borders of organisations are scarce, with a few exceptions.^21,22^ The Dutch questionnaire developed and studied by Berendsen *et al* quantifies how SCCs and GPs value their mutual collaboration. Nũno-Solinís *et al*’s Spanish questionnaire measures the conditions for collaboration, while taking the influence of the work environment into account.^21,22^

This article describes a study of the validity and test-retest reliability of Nũno-Solinís *et al*’s questionnaire in the Dutch healthcare context. The original questionnaire was developed based on a theoretical model for interprofessional collaboration by D’Amour.^22–25^ D’Amour described collaboration as 'the structuring of collective action through sharing of information and decision-making in clinical processes between professionals in different organisations,'^23–24^ and distinguished four dimensions of interprofessional collaboration. Two are relational dimensions: shared goals and vision, and internalisation; and two organisational dimensions: governance, and formalisation. Out of four dimensions, 10 interrelating indicators were formulated and evaluated in a multiple-case study^23–24^ (see Box 1).

**Box 1.** Dimensions and indicators of a conceptual model for interprofessional collaboration^23–24^

Factors	Dimensions	Indicators
**Interpersonal relationships**	Shared goals and vision	• Shared goals • Patient-centred orientation
	Internalisation	• Mutual acquaintanceship • Trust
**Organisational setting**	Governance	• Centrality • Leadership • Support for innovation • Connectivity
	Formalisation	• Formalisation tools • Information exchange

In Spain, these 10 indicators have been used in the development of a questionnaire which measures clinicians' perceptions about conditions for interprofessional collaboration.^22^ A study of the validity of the 10-item Spanish questionnaire showed a two-factor structure with promising levels of model fit.^22^ The present study aims to re-study the validity of the questionnaire developed in Spain, in order to test its appropriateness for measuring conditions for interprofessional collaboration in the Dutch healthcare setting. Data were collected in three geographic regions in the Netherlands where GPs and SCCs work in a hospital context to deliver collaborative patient care.

## Method

### Content validity and face validity

To test face-validity, 28 professionals were asked by mail to look at the questionnaire, translated from English to Dutch (full questionnaire available in English and Dutch from the authors, on request). These professionals (SCCs *n* = 20, GPs *n* = 8) were asked whether they could answer the questions and whether they considered the questions relevant for measuring collaboration in their working situation. Their main comment was about long sentences. In the subsequent translation from Spanish to Dutch, the length of the sentences was a major focus of attention.

The content validity was considered adequate because it was based on the diverse and thorough studies of D’Amour and the questionnaire-building process in Spain, together with the reactions of intended responders.

### Preparation of the questionnaire

Two bilingual translators forward-translated the original Spanish questionnaire into Dutch, and some adjustments were made to the questionnaire to take account of the Dutch healthcare setting. The structure of the 10-item Spanish questionnaire was preserved including, after each question, the phrase 'Please rate the current situation in your organisation with respect to the other level of care on a scale of 1 to 5.' The response options describe desirable attributes with five distinct descriptions for each item on a 5-point Likert scale, all ranging from 1 (none of the attribute) to 5 (lots of the attribute), as demonstrated in the example in Box 2. A backward translation from Dutch to Spanish was done. This translation into Spanish was reviewed and approved by one of the developers of the questionnaire.

**Box 2.** Example of two items of the 10–item questionnaire to assess the interprofessional collaboration of two different levels of care

**1. SHARED GOALS** **The existence of explicit shared goals facilitates collaboration and coordination between primary and secondary care settings.** **Please rate the current situation in your organisation with respect to the other level of care on a scale of 1 to 5:**				
1. Common goals are missing	2. There are hardly any shared goals	3.There are some common goals	4. There are quite a lot of common goals	5. Nearly all aspects of care are covered by shared goals

**2. PATIENT-CENTRED APPROACH** **When priority is given to the interests and preferences of the patient, this favours collaboration and coordination between professionals working in the primary and secondary care setting.** **Please rate the current situation in your organisation with respect to the other level of care on a scale of 1 to 5:**				
1. In the interaction between levels of care, the interests and preferences of patients are not taken into account	2. In the interaction between levels of care, the interests and preferences of patients are taken into account on few occasions	3. In the interaction between levels of care, the interests and preferences of patients are sometimes taken into account	4. In the interaction between levels of care, the interests and preferences of patients are often taken into account	5. In the interaction between levels of care, the interests and preferences of patients are always taken into account

After translation, the questionnaire was pre-tested, to check whether the translation introduced errors after the test for face validity, using the thinking aloud method.^25–26^ Pre-testing was done by five GPs (from city and urbanised rural areas) and five SCCs from different specialties, all native Dutch speakers. Each of them individually read each question aloud.^25–26^ A researcher then asked them how they interpreted the questions, and what they thought about the ease of comprehension. None of these professionals indicated relevant aspects of collaboration conditions that were missing in the questionnaire. All comments were noted and discussed within the research team. An example of change of a phrase is 'professionals working in the different levels' being changed to 'the primary and secondary care setting.'

After discussing all the comments in the team, a final version of the questionnaire was established.

### Data collection and sample

For validity testing, an invitation to complete the online questionnaire was sent to all GPs and SCCs (*N* = 1369) practising in one of three selected geographic regions in the Netherlands. Each of these regions contained one large hospital (a top, non-academic, clinical teaching hospital) with a central position in the region. Invitations were sent by e-mail with a link providing direct access to the questionnaire. In two regions, a reminder was sent to all non-responders 2 weeks after the first invitation. In the third region, all responders received the questionnaire again after 2 weeks to evaluate the test-retest reliability. The data were collected between October 2015 and March 2016.

### Data analysis

The approach to studying the (construct) validity of the translated questionnaire was similar to the approach used to study the validity of the original questionnaire.^22^ First, the construct validity of the questionnaire was examined using a CFA. Because of the ordinal structure of the data, this analysis was conducted by fitting the factor model on the polychoric correlation matrix^27^ of the item responses. The factor model is estimated by maximum likelihood on the data from the three regions separately, to avoid regional effects that may contaminate the correlation analyses and to explore the stability of the factor structure across the regions. Nũno-Solinís *et al*’s correlated two-factor model was assumed, with the uncorrelated error terms. Initially, measurement invariance across regions was not assumed. The fit of the confirmatory factor models was evaluated using common fit statistics: root mean square error of approximation, standardised root mean square residual, and comparative fit index, with usual cut-off points.

Next, an EFA with oblimin rotation (allowing the factors to be correlated) was employed, because the fit of the CFA models was insufficient. The approach taken was, again, similar to the EFA approach taken by Nũno-Solinís *et al*.^22^ The Bayesian Information Criterion (BIC) was used to determine the optimal number of factors.^28^ The lower the BIC, the better the fit of the factor model to the data.

Finally, test-retest reliability was evaluated on item-level by squared weighted Kappa (SW Kappa) statistics,^29^ taking an SW Kappa of >0.70 as the threshold criterion for reliability.

All analyses were performed in R (version 3.1.1.) using the Lavaan package (CFA), Psych package (EFA), and *irr* package (Kappa).

## Results

### Response

From the 1369 (582 GPs and 787 SCCs) invitees, 458 doctors responded: 206 GPs and 252 SCCs. Thirteen questionnaires were incomplete. There was a total of 445 fully completed questionnaires, representing a response rate of 33% (Table 1).

**Table 1.** Response rate in three Dutch regions

	Total sent	Responses, *n*	Response rate, %
Region 1	587 (334 GPs, 253 SCCs)	203	35
Region 2	398 (249 GPs, 149 SCCs)	84	21
Region 3	384 (204 GPs, 180 SCCs)	158	41
**Total**	**1369 (787 GPs, 582 SCCs)**	**445**	**33**

SCC = secondary care clinician.

### CFAs

The CFA explored the two-factor structure of the questionnaire (interpersonal relationships and organisational setting) in the Dutch healthcare setting, as assumed and tested by Nuño-Solinís *et al*.^22^ The fit indices, with their corresponding suggested thresholds levels for sufficient fit to the data, are listed in Table 2. None of the fit indices met the norm criteria for a sufficient fit in each of the three regions.

**Table 2.** Fit indices for the questionnaire in the Netherlands, by region

Fit indices	Threshold for sufficient fit	Region 1 (*n* = 203)	Region 2 (*n* = 84)	Region 3 (*n* = 158)
Root mean square error of approximation	<0.08	0.14	0.17	0.15
Standardised root mean square residual	<0.08	0.08	0.09	0.09
Comparative fit index	>0.90	0.79	0.72	0.66

**Figure 1:**
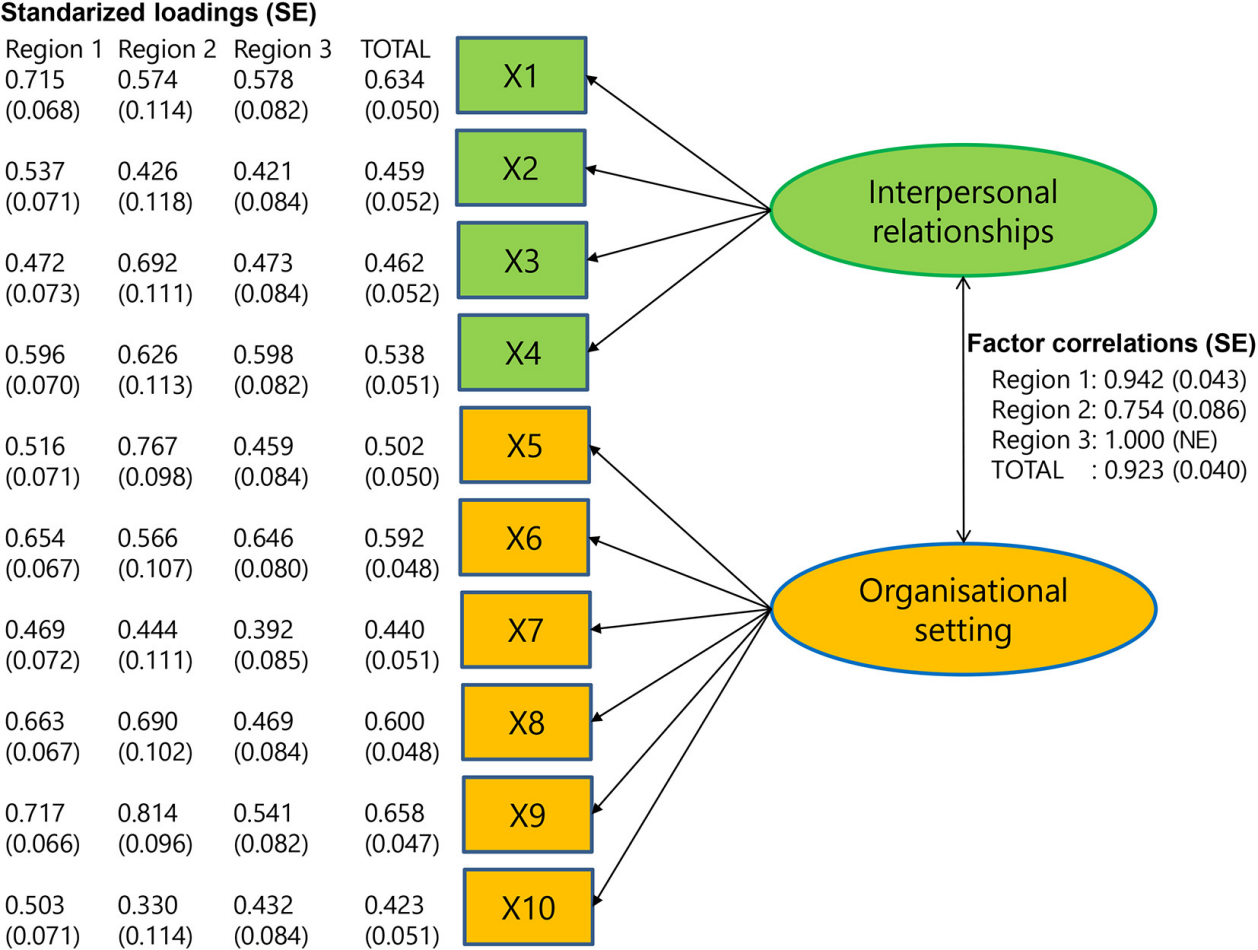
CFA diagram. Results of the confirmatory factor analysis in three regions and the total. NE = not estimable. SE = standard error.

The questions X1–X4 aimed to measure the factor 'interpersonal relationships' and the questions X5–X10 aimed to measure 'organisational setting'. Figure 1 details the estimated CFA diagram^30^ assuming a two-factor structure, the estimated standardised loadings, and corresponding maximum likelihood standard errors. The estimated factor loadings show notable variation between regions and items. High factor loadings (>0.7) were rare (*n* = 4/30). The estimated factor correlations are 0.942 (region 1), 0.754 (region 2), 1.000 (region 3), and 0.923 (total). In all, the estimated factor structures varied considerably between the three regions and, in each of the regions, a lack of support was shown for the assumed two-factor structure. Exploration of modification indices (that is, suggestions for model improvements, such as allowing correlation of error variances) did not yield consistent suggestions for improvements that could improve model fit across the regions (results not shown).

### EFAs

To explore alternative factor structures, EFAs were carried out. EFA models with one, two, three, four, five, and six factors were fitted for each of the regions. Similar to the CFA, the EFA with oblimin rotation showed unstable factor structures across regions. Based on the BIC, a four-factor structure was found to be optimal in region 1; five-factor in region 2; and another five-factor in region 3 (other criteria to determine the optimal number of factors were also checked but did not resolve the instability). The factor loadings for the four and five-factor models were not stable across regions (results not shown). Further explorations with alternative orthogonal rotation strategies and factor selection criteria did not provide a solution to the instability of the factor structure.

### Test-retest reliability

Test-retest reliability analyses were performed with the data gathered in region 3 (*N* = 90, GPs = 51, SCCs = 39). The test-retest was measured with the SW Kappa agreement, and is shown in Table 3. The test-retest reliability of this questionnaire was shown to be insufficient for each item (SW Kappa >0.70 is sufficient).

**Table 3.** Test-retest reliability, region 3 (*N* = 90), measured with the SW Kappa^a^. Confidence intervals were estimated by non-parametric bootstrap procedure, based on 5000 bootstrap samples

Questions	Items	SW Kappa	95% CI
X1	Shared goals	0.39	0.27 to 0.50
X2	Patient-centred approach	0.63	0.43 to 0.79
X3	Mutual knowledge	0.42	0.17 to 0.61
X4	Trust	0.43	0.25 to 0.60
X5	Strategic guidelines	0.36	0.22 to 0.48
X6	Shared leadership	0.41	0.20 to 0.58
X7	Support for innovation	0.31	0.07 to 0.52
X8	Forums for meeting	0.56	0.41 to 0.69
X9	Protocolisation	0.32	0.19 to 0.44
X10	Information systems	0.54	0.37 to 0.67

^a^SW Kappa >0.70 the threshold criterion for reliability. SW Kappa = squared weighted Kappa.

## Discussion

### Summary

In the present study, an evaluation was conducted of the validity and test-retest reliability of a carefully translated and culturally-adapted questionnaire, which aimed to measure interprofessional collaboration conditions between clinicians at different levels of care. In contrast to the original questionnaire, the questionnaire showed poor validity in the Dutch setting, and the additional test-retest reliability was also insufficient. The data collected in three different Dutch regions allowed for a cross-regional comparison of validity, by comparing confirmatory factor models with sufficient sample sizes in each region. This study provides clear evidence that the hypothesised two-factor structure that underlies this questionnaire cannot be confirmed in any of the regions. Consequently, the authors advise against the use of this questionnaire to measure conditions for interprofessional collaboration in the Dutch healthcare context.

One explanation for the difference between validity of the Spanish and Dutch questionnaire may be the differences in organisation of the healthcare system. In the Spanish region where the questionnaire was validated, clinical professionals (doctors and nurses) are part of one integrated care delivery organisation, consisting of a regional hospital (specialised care level), and health centre (primary care level), and are contracted with one provider. In the Netherlands, primary and secondary care takes place in separate organisations and is contracted with different providers. Dutch primary care consists of organisations varying in form and size. Between the Dutch regions, cultural and organisational differences exist and the questionnaire is not stable in its two-factor structure. Different factors were found with the EFA for the regions. The questionnaire appears to be culturally-sensitive.

### Strengths and limitations

A strength of this study is that statistical analyses were carried out on a larger sample than has been used in Spain. The questionnaire has been tested in three different Dutch regions, and hence was applied in different organisational circumstances within the healthcare system. The analyses were performed by region and can be perceived as three separate studies. The sample size in one of the regions was smaller than recommended.^30^ In the other regions, a sample size was obtained that was close to the recommended sample size. Finally, the analysis of the remaining region and the analysis on the entire data set were well above the recommended sample size. Another strength of this study is the measurement of the test-retest reliability. To test whether measurement results are reproducible in test–retest situations is important.^28,31^ No test-retest reliability analysis had been conducted for the original questionnaire. A possible explanation of the poor test-retest results could be current collaborative experiences influencing the opinions of the responding medical professionals.

A limitation of this study is that the response rate (33%), although higher than in the original study, was limited.

### Comparison with existing literature

The strong theoretical base of the questionnaire, which was derived from several qualitative studies by D’Amour *et al*, supports the importance of the 10 dimensions related to interprofessional collaboration.^5,24,27^ But to measure the relational and organisational items with a questionnaire comprising 10 questions seems difficult. One study which measured how GPs and SCCs rate their collaboration did not take into account the organisation they worked in.^21^

### Implications for research and practice

Even though the surveyed healthcare professionals considered the questions based on these dimensions relevant for their work in the Dutch healthcare context, the questionnaire proved not to be valid in the Netherlands. The construct validity and test-retest reliability of this translated questionnaire were insufficient to warrant use in the Dutch setting. To develop a useful questionnaire about collaboration, more questions about each of the indicators are probably needed, which means constructing a new questionnaire and testing it with an approach similar to the one presented here. Alternatively, use of qualitative methods such as interviews could lead to more insight into interorganisational collaboration. Even though a pre-testing process might indicate a high face-validity, it is not a guarantee as to other aspects of validity. Even between regions in the same country, cultural differences can exist.

Importantly, this study demonstrates that even when the face-validity of a translated questionnaire seems to be good, thanks to a pre-testing process with sufficient opportunities for cultural adaptation, other aspects of validity must be tested before applying the instrument in new settings. Even between regions, there can be cultural differences. The authors advise caution when using a questionnaire in another cultural setting, population, or context without testing it. Before choosing a questionnaire for a small-scale study in general practice, the authors recommend checking that it is validated for that setting.

## References

[1] Barnett K, Mercer SW, Norbury M (2012). Epidemiology of multimorbidity and implications for health care, research, and medical education: a cross-sectional study. Lancet.

[2] Cassel CK, Reuben DB (2011). Specialization, subspecialization, and subsubspecialization in internal medicine. N Engl J Med.

[3] Institute of Medicine (US) Committee on Quality of Health Care in America (2001). A New Health System for the 21st Century: Crossing the Quality Chasm.

[4] Starfield B (2010). Primary care, specialist care, and chronic care: can they interlock?. Chest.

[5] D'Amour D, Ferrada-Videla M, San Martin Rodriguez L (2005). The conceptual basis for interprofessional collaboration: core concepts and theoretical frameworks. J Interprof Care.

[6] Göbel B, Zwart D, Hesselink G (2012). Stakeholder perspectives on handovers between hospital staff and general practitioners: an evaluation through the microsystems lens. BMJ Qual Saf.

[7] Smith SM, Allwright S, O'Dowd T (2007). Effectiveness of shared care across the interface between primary and specialty care in chronic disease management. Cochrane Database Syst Rev.

[8] Akkerman SF, Bakker A (2011). Boundary crossing and boundary objects. Review of Educational Research.

[9] Hesselink G, Vernooij-Dassen M, Pijnenborg L (2013). Organizational culture: an important context for addressing and improving hospital to community patient discharge. Med Care.

[10] Yemm R, Bhattacharya D, Wright D (2014). What constitutes a high quality discharge summary? A comparison between the views of secondary and primary care doctors. Int J Med Educ.

[11] Johnson JK, Farnan JM, Barach P (2012). Searching for the missing pieces between the hospital and primary care: mapping the patient process during care transitions. BMJ Qual Saf.

[12] Kripalani S, Jackson AT, Schnipper JL (2007). Promoting effective transitions of care at hospital discharge: a review of key issues for hospitalists. J Hosp Med.

[13] O'Malley AS, Reschovsky JD (2011). Referral and consultation communication between primary care and specialist physicians: finding common ground. Arch Intern Med.

[14] Berendsen AJ, de Jong GM, Meyboom-de Jong B (2009). Transition of care: experiences and preferences of patients across the primary/secondary interface — a qualitative study. BMC Health Serv Res.

[15] Hassink-Franke LJ, Janssen MM, Oehlen G (2016). GPs' experiences with enhanced collaboration between psychiatry and general practice for children with ADHD. Eur J Gen Pract.

[16] Chiocchio F, Lebel P, Dubé JN (2016). Informational role self-efficacy: a validation in interprofessional collaboration contexts involving healthcare service and project teams. BMC Health Serv Res.

[17] Goldman J, Reeves S, Wu R (2016). A sociological exploration of the tensions related to interprofessional collaboration in acute-care discharge planning. J Interprof Care.

[18] Bosch M, Dijkstra R, Wensing M (2008). Organizational culture, team climate and diabetes care in small office-based practices. BMC Health Serv Res.

[19] Orchard CA, King GA, Khalili H (2012). Assessment of Interprofessional Team Collaboration Scale (AITCS): development and testing of the instrument. J Contin Educ Health Prof.

[20] Ohman-Strickland PA, John Orzano A, Nutting PA (2007). Measuring organizational attributes of primary care practices: development of a new instrument. Health Serv Res.

[21] Berendsen AJ, Benneker WH, Groenier KH (2010). DOC questionnaire: measuring how GPs and medical specialists rate collaboration. Int J Health Care Qual Assur.

[22] Nuño-Solinís R, Berraondo Zabalegui I, Sauto Arce R (2013). Development of a questionnaire to assess interprofessional collaboration between two different care levels. Int J Integr Care.

[23] D'Amour D, Goulet L, Labadie JF (2008). A model and typology of collaboration between professionals in healthcare organizations. BMC Health Serv Res.

[24] D'Amour D, Oandasan I (2005). Interprofessionality as the field of interprofessional practice and interprofessional education: an emerging concept. J Interprof Care.

[25] Oandasan I, D’Amour D, Zwarenstein M (2004). Interdisciplinary education for collaborative, patient-centred practice research and findings report.

[26] World Health Organization (2018). Process of translation and adaptation of instruments. http://www.who.int/substance_abuse/research_tools/translation/en/.

[27] Olsson U (1979). Maximum likelihood estimation of the polychoric correlation coefficient. Psychometrika.

[28] Fraley C (1998). How many clusters? Which clustering method? Answers via model-based cluster analysis. The Computer Journal.

[29] Fleiss JL, Cohen J (1973). The equivalence of weighted kappa and the intraclass correlation coefficient as measures of reliability. Educ Psychol Meas.

[30] Kline RB (2005). Principles and practice of structural equation modelling.

[31] Beatty PC, Willis GB (2007). Research synthesis: The practice of cognitive interviewing. Public Opin Q.

